# The Genomic Physics of COVID-19 Pathogenesis and Spread

**DOI:** 10.3390/cells11010080

**Published:** 2021-12-28

**Authors:** Ang Dong, Jinshuai Zhao, Christopher Griffin, Rongling Wu

**Affiliations:** 1 Center for Computational Biology, Beijing Forestry University, Beijing 100083, China; fantasys05227@163.com (A.D.); zjs619938@163.com (J.Z.); 2 Applied Research Laboratory, The Pennsylvania State University, University Park, PA 16802, USA; cxg286@psu.edu; 3 Center for Statistical Genetics, Departments of Public Health Sciences and Statistics, The Pennsylvania State University, Hershey, PA 17022, USA

**Keywords:** COVID-19, person-to-person transmission, horizontal epistasis, hypergraph, genetic loci

## Abstract

Coronavirus disease (COVID-19) spreads mainly through close contact of infected persons, but the molecular mechanisms underlying its pathogenesis and transmission remain unknown. Here, we propose a statistical physics model to coalesce all molecular entities into a cohesive network in which the roadmap of how each entity mediates the disease can be characterized. We argue that the process of how a transmitter transforms the virus into a recipient constitutes a triad unit that propagates COVID-19 along reticulate paths. Intrinsically, person-to-person transmissibility may be mediated by how genes interact transversely across transmitter, recipient, and viral genomes. We integrate quantitative genetic theory into hypergraph theory to code the main effects of the three genomes as nodes, pairwise cross-genome epistasis as edges, and high-order cross-genome epistasis as hyperedges in a series of mobile hypergraphs. Charting a genome-wide atlas of horizontally epistatic hypergraphs can facilitate the systematic characterization of the community genetic mechanisms underlying COVID-19 spread. This atlas can typically help design effective containment and mitigation strategies and screen and triage those more susceptible persons and those asymptomatic carriers who are incubation virus transmitters.

## 1. Gene Networks as a Driver of Interpersonal Variability and Transmissibility

The atypical pneumonia COVID-19 is caused by a novel coronavirus, namely severe acute respiratory syndrome coronavirus 2 (SARS-CoV-2), and has been sweeping the globe [1,2,3,4]. An increasing number of studies have begun to unravel the molecular- and even atomic-level mechanisms for human–virus interactions through sequencing and structural analysis [5,6,7]. These studies have been instrumental in identifying the receptors, such as ACE2, the SARS-CoV2 virus uses to penetrate human cells and the key pathways through which the viral trimeric spike protein binds to host receptors [7,8,9]. However, the development of specific therapeutics to eradicate COVID-19 from these results may be impaired by two unsolved issues. First, existing approaches can only identify individual key genes, proteins, or metabolites associated with COVID-19 infection [10,11,12,13,14], but ample evidence shows that a complex disease involves a web of interactions among different genes [15,16]. Second, there is great variability in the number of receptors among individual hosts, which leads to high interpersonal heterogeneity in COVID-19 infection and symptoms [17,18]. For example, SARS-CoV-2 affects certain individuals more than others under the same circumstances [19]. Some individuals are more susceptible to, but not necessarily more infected by, the viruses than others [20,21]. Many research consortia and pharmaceutical sectors have begun to sequence both human and SARS-CoV-2 genomes in a quest to understand the genetic basis of COVID-19 transmission [22,23,24,25,26].

We argue that these two issues can be disentangled by reconstructing interaction networks that coalesce all genes, proteins, metabolites, and DNA variants into mathematical graphs. There are two types of such networks, one inferred from genomic data (transcriptomic, proteomic, and metabolomic) and the other from genetic data (DNA genotype). The surge of network reconstruction in the last two decades has attracted the development of a number of computational models and methods for a wide range of disciplines [27,28,29]. However, a majority of existing methods can only identify an overall network from a number of samples but cannot characterize sample-specific variation. Additionally, these methods can only capture either the strength of interaction or the direction of interaction, failing to combine these network properties [30,31]. More recently, Wu and Jiang [32] proposed a generic model for reconstructing fully informative networks that code the strength of interactions, bicausality of links, and the sign of causality, as defined in Chen et al. [33]; one salient advantage of this approach is the recovery of dynamic networks from static data. All approaches are developed based on omics expression data collected from each sample, but they are not adjusted to consider DNA variant data in which each sample is represented by a genotype, associated with a phenotypic value.

In this article, we extend Wu and Jiang’s network model to reconstruct COVID-19-induced genomic networks from static expression data. Based on the transmission behavior of epidemics, we integrate quantitative genetic principles into graph theory to reconstruct mobile networks that trace and monitor the genetic architecture of COVID-19 spread in human communities. We give examples of how the models described can be applied to quantify the topological changes of genomic interactions from a healthy state to a diseased state. Simulation studies are performed to examine the statistical properties of the models.

## 2. A Physical Model for Contextualizing Genomic Networks

To characterize the molecular mechanisms underlying COVID-19 pathogenesis, Leng et al. [10] monitored and compared proteomic profiles in SARS-CoV-2-infected lung tissues and healthy lung tissues. Although key differentially expressed proteins have been identified in response to SARS-CoV-2 infection, it is likely that the occurrence of the disease is not only mediated by these individual proteins but also through the complex interactions of all proteins. We modify Wu and Jiang’s model to accommodate Leng et al.’s sampling strategy that measured 3220 proteins from three COVID-19-infected (but healthy prior to infection) patients and eight controls without COVID-19 pneumonia, totaling 11 samples.

### 2.1. The Integration of Allometric Scaling Law and Evolutionary Game Theory

**Figure 1:**
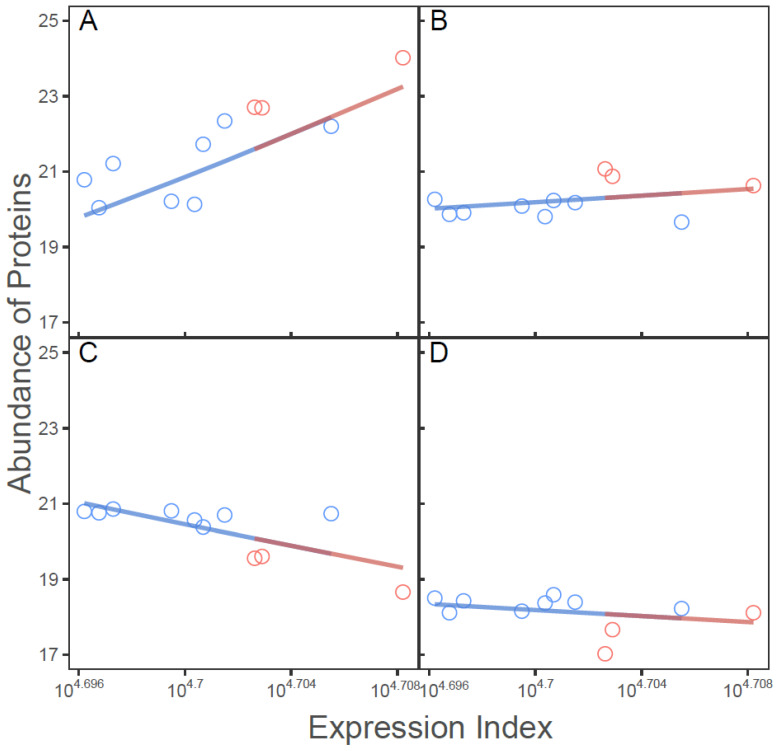
Allometric scaling fitting of abundance of individual proteins to expression index across 11 samples (eight healthy lungs, cold-color dots; and three SARS-CoV-2-infected lungs, warm-color dots). Four representative proteins, P0DOX7 (**A**), GAPDH (**B**), PPIA (**C**), and RPLP2 (**D**) are chosen.

Let *y_ij_* denote the abundance of protein *j* (*j* = 1, …, 3220) on sample *i* (*i* = 1, …, 11). The total amount of abundance of all proteins for each sample is calculated and defined as an expression index, denoted by $E_{i}=\overset{3220}{\underset{j=1}{\sum }}y_{ij}$. Thus, *y_ij_* and *E_i_* establish a part–whole relationship that obeys the allometric scaling law described by a power equation [34,35]. Figure 1 illustrates examples of allometric scaling relationships for four randomly chosen proteins. We find that some proteins, e.g., PoDOX7 (immune one strand of globulin, served as receptors that trigger the clonal expansion and differentiation of B lymphocytes into immunoglobulins-secreting plasma cells) (Figure 1A) and glyceraldehyde-3-phosphate dehydrogenase (GAPDH, which catalyzes an important energy-yielding step in carbohydrate metabolism) (Figure 1B) increase their abundance with expression index, but to different extents, whereas the abundance of others, such as peptidylprolyl isomerase A (PPIA, which catalyzes the cis-trans isomerization of proline imidic peptide bonds in oligopeptides) (Figure 1C) and ribosomal protein lateral stalk subunit P2 (RPLP2, which plays an important role in the elongation step of protein synthesis) (Figure 1D), decreases with expression index. It is interesting to note that the total expression level of all proteins--expression index--is higher in SARS-CoV-2-infected lungs than healthy lungs. Taken together, the abundance of individual proteins measured once on each sample, in spite of its static nature, can be expressed as a “dynamic” function of expression index. Tremendous variability in the form of such a function implies the occurrence of protein–protein interactions across samples.

We integrate evolutionary game theory [36] to interpret how individual proteins change abundance with expression index through their interactions and interdependence with other proteins. This theory allows us to assume that all proteins form a system in which the expression of any one protein is determined by its own “strategy” and the strategies of other proteins that interact with it. To quantify the dynamic behavior of the system based on evolutionary game theory, we introduce the allometric scaling law to develop a system of ordinary differential equations, expressed as

(1) $$y_{j}^{\prime }\left(E_{i}\right)=Q_{j}\left(y_{j}\left(E_{i}\right);\phi_{j}\right)+\overset{p}{\underset{j^{\prime }=1,j^{\prime }\neq j}{\sum }}Q_{j\leftarrow j^{\prime }}\left(y_{j^{\prime }}\left(E_{i}\right);\phi_{j\leftarrow j^{\prime }}\right)$$

where $Q_{j}\left(y_{j}\left(E_{i}\right);\phi_{j}\right)$ describes the (independent) expression level of protein *j* when it is assumed to be in isolation, and $Q_{j\leftarrow j^{\prime }}\left(y_{j^{\prime }}\left(E_{i}\right);\phi_{j\leftarrow j^{\prime }}\right)$ describes the (dependent) expression level of protein *j* regulated by protein $j^{\prime }$. The ODEs of Equation (1) are called quasidynamic ODEs (qdODEs) because their time derivative is replaced by the expression index derivative [33,37].

We fit the independent and dependent expression levels across samples using a nonparametric approach and estimate the ODE parameters by implementing the fourth-order Kutta–Runge algorithm. The estimated dependent expression levels of each protein regulated by other proteins are encapsulated into a mathematical network, filled with bidirectional, signed, and weighted interactions. Because dependent expression levels are a function of the expression index, we can reconstruct sample-specific networks. We further assemble eight sample-specific networks into a control network and three sample-specific networks into a SARS-CoV-2-infected network. Key links that determine differences between these two networks are regarded as the determinants of disease and healthy states.

### 2.2. Modularity Theory and Dunbar’s Law

**Figure 2:**
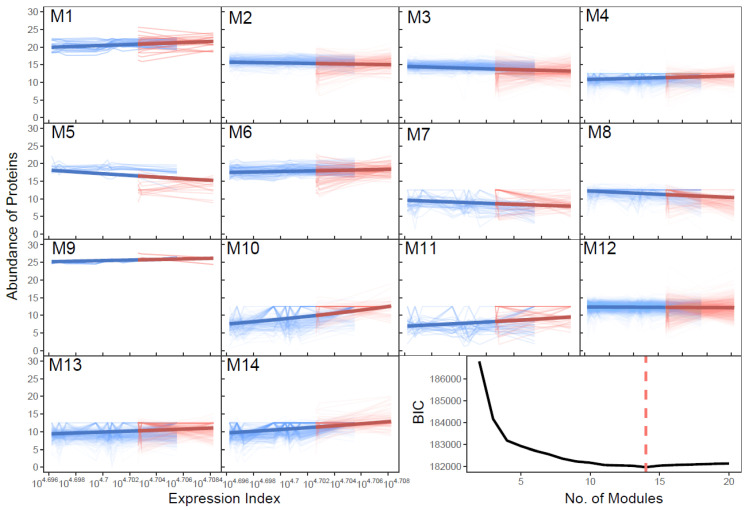
Fourteen distinct modules (labeled as M1–M14) identified among 3220 proteins based on their allometric scaling relationship with expression index according to BIC. Each thin line represents a protein within a module whose mean fitting is denoted by a thick line. Bluer lines and redder lines represent eight healthy lungs and three SARS-CoV-2-infected lungs, respectively [10].

It is not possible that all 3220 proteins are fully interconnected, because a full network is not robust enough to buffer against stochastic perturbations [38,39,40,41]. Instead, a large network should be modular and sparse to maintain the stability and robuestness of the complex system. A system is always heterogeneous, occurring as the consequence of dynamic interactions between modules. A module is defined as a relatively homogeneous area that differs from its surroundings in terms of the function of constituent elements [42,43]. This modularity theory allows us to classify all proteins into distinct modules based on the similarity of expression index-varying expression profiles. Because of this similarity, proteins in a module are more tightly connected with each other than with those from different modules. According to network theory, these different modules form network communities that play a different role in the overall behavior of the system. Functional clustering is an algorithm that can sort elements into different categories according to how elements behave as a function of time [44,45]. We implement power equation-based functional clustering to break down a 3220-node large network into well-delimited network communities. BIC analysis shows that the optimal number of such network communities is 14, each displaying a different dynamic pattern of protein expression over samples (Figure 2). SARS-CoV-2-infected lungs tend to display a higher expression index than healthy lungs, thus the expression index may roughly serve as a biomarker of COVID-19 infection.

In a primatological study, Dunbar [46] noted that there is a cognitive limit to the number of individuals with whom a primate can maintain stable social relationships. Dunbar explained this limit to be imposed by the neocortical processing capacity of primates. This so-called Dunbar’s law has been applied in many different fields, including evolutionary psychology, statistics, and business management [47,48]. If Dunbar’s law occurs in gene regulatory networks, this implies that the number of other proteins each protein can “recognize” (or sensibly interact with) is limited, causing sparsity in the network structure. Based on a regression model built from qdODEs of Equation (1), we implement regularization-based variable selection to choose a small set of proteins that are significantly associated with each protein [49]. This approach (in particular) can handle the issue of the curse of dimensionality, i.e., that the number of proteins is much larger than the number of samples. By incorporating the most significant proteins chosen for a given protein into qdODEs of Equation (1), we rederive a system of reduced equations from which to reconstruct sparse networks.

### 2.3. SARS-CoV-2-Induced Network Change

**Figure 3:**
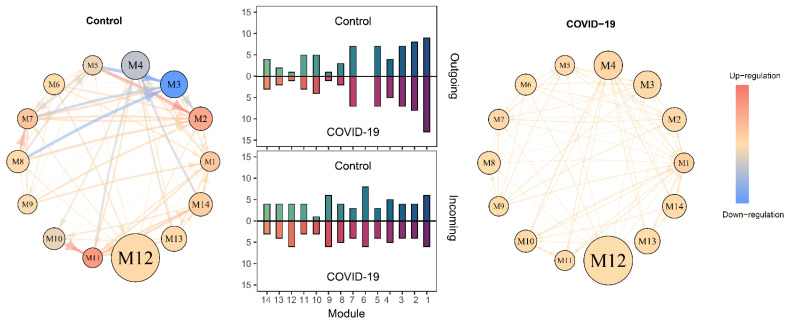
Multilayer protein–protein interaction networks for healthy (control) and SARS-CoV-2-infected lungs. Coarse-grained networks where nodes are modules (with the size of circles proportional to the total abundance of proteins in the modules) and edges are interactions between pairs of proteins (warm-color and cold-color line arrows indicate the promotion and inhibition, respectively, with link thickness proportional to the strength of interaction). Plots in the middle are the distribution of outgoing links and incoming links over different modules.

As described above, a large-scale network composed of 3220 proteins contains 14 network communities. Using the mean expression levels of all proteins within the same modules, we reconstruct 14-node intermodule networks coalescing network communities, called coarse-grained networks, for individual samples. We convert sample-specific networks into a healthy coarse-grained and a diseased coarse-grained network as a biomarker of SARS-CoV-2 infection (Figure 3). A link in a network is said to be outgoing or incoming if one node affects or is affected by the other node. Healthy and diseased networks have a similar structure of interactions, as revealed by their similar distribution of outgoing and incoming links over different modules. Yet, the two networks differ dramatically in topological organization (Figure 3). In general, the healthy network displays stronger interactions than the COVID-19 network, suggesting that protein–protein crosstalk becomes weak after lungs are infected by SARS-CoV-2.

**Figure 4:**
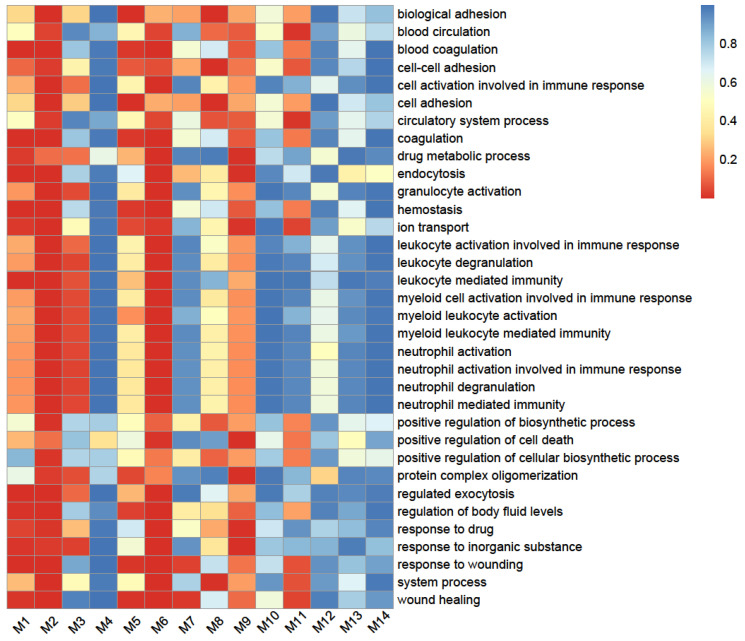
GO enrichment analysis of proteins from each module in terms of biological proteins. The color metric of each rectangle represents the *p* value of corresponding GO terms, with red approximately 0 (significant) and blue approximately 1 (not significant). The map was made using R package pheatmap.

Module M3 is a key module whose links well distinguish the healthy network from the disease network (Figure 3). GO enrichment analysis shows that proteins involved in this module have a major function related to the activation of neutrophil, myeloid, leukocytic, and granulocytic cells that affects immune response (Figure 4). Based on the coarse-grained networks (Figure 3), we postulate that the immune system of healthy individuals (determined by M3) is inhibited by the proteins with functions in cell and biological adhesion (Module M8) and wound healing (Module M7), but during SARS-CoV-2 infection, the immune system is jointly activated by various types of proteins.

To further explore how COVID-19 induces the abundance change of proteins as a whole, we reconstruct fine-grained networks filled with interactions expressed at the protein level. As an example, we choose module M3 that was identified to mediate the immunity system of humans. This module contains 463 proteins that form a web of interactions among its proteins, and from this web, a clear roadmap of how each protein interacts with every other protein can be characterized (Figure S1). In general, the interaction networks of these proteins are sparse, displaying a similar structure for both healthy and diseased individuals. The difference between the healthy and COVID-19 networks lies in the strength of protein–protein interactions. For example, DDX39B inhibits the expression of PSMB9 for healthy individuals, but the extent of this inhibition is dramatically reduced for SARS-CoV-2-infected individuals. On the other hand, the promotion of FBLN6 by CD9 is reduced when healthy individuals become infected. The differences in these interactions and other interactions may be a determinant of COVID-19 infection and noninfection. It should be pointed out that our conclusion is based on a modest sample size (*N* = 11), whose more convincing interpretation relies on a larger sample size (say 50 or more) [32]. Yet, our study provides a starting point to more precisely explore the genomic signature of COVID-19 from informative gene regulatory networks.

## 3. Statistical Genetic Physics of COVID-19 Spread

As an infectious disease, COVID-19 shows strong person-to-person transmissibility, whose basic estimated reproductive number is as high as about 2.68 (95% CrI 2.47 to 2.86) [3]. Because of this, the efficient and effective control of this disease requires a detailed understanding of the community machinery that mediates transmission and spread. Interpersonal variability has been observed in the pattern and speed at which COVID-19 transmits from person to person in communities. Despite a high number of contacts (372), the first known person-to-person transmission cases did not result in transmission of the virus [50,51]. In another case, an asymptomatic carrier has been shown to transmit the virus to five family members [21]. These findings show that viruses from some carriers are more transmissible than those from others. Indeed, well-controlled epidemiological studies using animal models also documented this phenomenon [51,52], regarded as being universal in infectious disease contagions. Like the effects they exert on disease severity, genes can also play a critical role in determining interpersonal transmissibility. Conventional genetic analyses can dissect the interpersonal variability of a disease, but they have no power to characterize the genetic mechanisms underlying the interpersonal transmissibility of the disease as a dynamic process.

We argue that the COVID-19 pandemic is determined by a genetic system composed of genes from a transmitter, recipient, and viruses. Genes from the transmitter interact with those from the recipient to affect the COVID-19 severity of the recipient, and, meanwhile, the sign and strength of these interactions are mediated by SARS-CoV-2 genes. We propose a computational framework for quantitatively coalescing transmitter–recipient–virus interactions, pertaining to the genetic system into a hypergraph. Subsequent transmissions of the virus to other individuals lead to the formation of a new genetic system. Our mobile hypergraphs can capture such dynamic changes, equipped with a capacity to decipher how COVID-19 spreads from person to person through close contacts.

### 3.1. Horizontal Epistasis: An Emerging Concept

Darwin’s evolutionary theory suggests that the phenotype of an individual affects the phenotypes of other individuals in the same community to an extent that drives phenotypic variation and evolution [53,54,55]. Quantitative genetic studies of this phenomenon indicate that the phenotype of one individual is not only determined directly by their own genes but also indirectly by the genes of individuals with whom it co-exists, in an epistatic fashion [56,57,58]. In an association study, Biscarini et al. [59] identified a number of loci that exert indirect genetic effects on plumage conditions in laying hens. Genes with indirect genetic effects were also identified to affect size, development, and fitness traits in *Arabidopsis* [60]. Relative to vertical epistasis, described as the effect of the interactions between genes from different genomic locations within the same individual [61], we define the interaction effects of genes across different individuals as horizontal epistasis. Faced with a viral invasion, a recipient will activate innate and adaptive immune responses through certain genes, e.g., those within the major histocompatibility complex (MHC) locus, to produce specific antibodies that coat viruses and reduce their infection [62,63], during which the virus will evolve specific strategies, including mutations or methylations, to evade these responses or adapt to the new environment of the recipient [64,65,66]. The pattern and degree of the mutation or the epigenetic alteration of viral genes depend on how they interact with the physiological environment of the recipient. Thus, when this recipient becomes a transmitter, their “personalized” viruses will preferentially attack the next recipient who can provide the essential environment for the viruses to survive. In the case of person-to-person transmission, the viruses serve as a genetic “messenger” that links the transmitter to the recipient unidirectionally and, thus, are regarded as a stimulus that elicits horizontal epistasis. This pattern of interpersonal transmission is essentially the consequence of the joint influences of genes from the transmitters, recipients, and viruses. To systematically characterize the genetic mechanisms underlying the rate and intensity of SARS-CoV-2 spread, we need to chart the network of gene interactions among these three parties. In some situations, viruses spread from natural hosts (e.g., animals) to alternative hosts (humans) to form a more complex pandemic network [67]. Revealing the mechanisms that lie behind this network includes the characterization of horizontal epistasis among genes from humans, animals, and viruses.

Graph theory has been widely used as a tool to reconstruct gene networks [68,69]. This approach can only characterize pairwise interactions coded as edges of the graph, with each edge adjacent (connecting) to two nodes. However, the process of person-to-person or animal-to-person transmissions includes genes from more than two genomes and, thus, high-order horizontal interactions are likely to trigger their effects. Next, we show that a hypergraph, the generalization of a graph to allow an edge to join more than two nodes [70,71], can precisely capture how genes from different genomes interact globally with each other to determine the spread of COVID-19.

### 3.2. Genetic Hypergraphs

**Figure 5:**
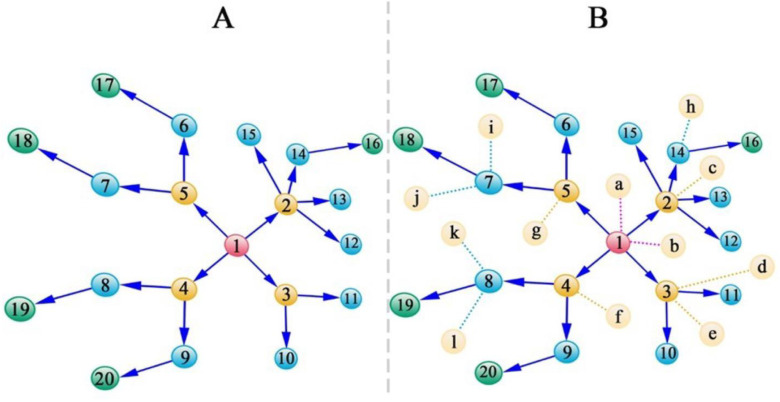
Transmission path *of* SARS-CoV-2 through contacts. (**A**) A strategy for sampling transmitters and recipients, the direction of whose transmission is denoted by arrows. (**B**) A strategy for sampling transmitters and recipients, the direction of whose transmission is denoted by arrows, as well as individuals who are not infected (labeled by letters) even after contact with the transmitters. Numbers in yellow refer to those that transmit the virus to multiple recipients. In a graph, these transmitters are likely to be regarded as hub nodes.

SARS-CoV-2 spreads mainly through person-to-person contacts [1,2,3,4], where routes of interpersonal transmission can be retroactively tracked by recording and monitoring contact history [72]. A recipient is assumed to be connected to only one transmitter, but a transmitter may have multiple recipients. Let us imagine a spread path as illustrated in Figure 5A, where the first transmitter transforms the virus to Recipients 2, 3, 4, and 5, who then become transmitters for subsequent recipients. Now, let us take a step further and assume that we can sequence the genomes of these affected individuals, measure their pneumonia-related clinical outcomes, and also screen the genome-wide haploid (epi)genetic alterations of the viruses inhabiting each recipient.

Hypergraphs have been increasingly recognized as a powerful tool to model complex systems, such as cell–cell interactions [71] and epidemic propagation [73]. Here, we develop a genetic version of hypergraphs to model genome–genome interactions. During the SARS-CoV-2 spread, a transmitter passes on the virus to a recipient, forming a small functional triad unit composed of three entities. We argue that such a unit forms a hypergraph in which genes from three genomes represent nodes, pairwise cross-genome interactions define edges, and three-order cross-genome interactions define hyperedges. This interactive unit propagates COVID-19 along reticulate paths to spread into communities. In this sense, the scope of COVID-19 spread can be dissected into a series of dynamically interconnected units. We integrate quantitative genetic theory and hypergraph theory to quantify the pattern and strength of various cross-genome interactions, i.e., horizontal epistasis, displayed in each unit.

Consider a sample of infected individuals from a human population. There are three diploid genotypes at a human single-nucleotide polymorphism (SNP) locus with two alleles and two haploid genotypes at a virus locus. Three transmitter genotypes, three recipient genotypes, and two virus genotypes are randomly combined to form 18 three-way genotype combinations. There is variability in the clinical outcomes of recipients among these combinations. We can partition the genotypic value of a combination for a disease outcome/phenotype, e.g., pneumonia severity, into the following components:

- Direct main effects of the gene of the recipient on its own phenotype;
- Indirect main effects of the gene of the transmitter on the phenotype of the recipient;
- Indirect genetic effect of the virus gene on the phenotype of the recipient;
- Horizontal two-way epistatic effects between the transmitter gene and recipient gene on the phenotype of the recipient;
- Horizontal two-way epistatic effects between the virus gene and transmitter gene on the phenotype of the recipient;
- Horizontal two-way epistatic effects between the virus gene and recipient gene on the phenotype of the recipient;
- Horizontal three-way epistatic effects among the virus gene, transmitter gene, and recipient gene on the phenotype of the recipient.

**Figure 6:**
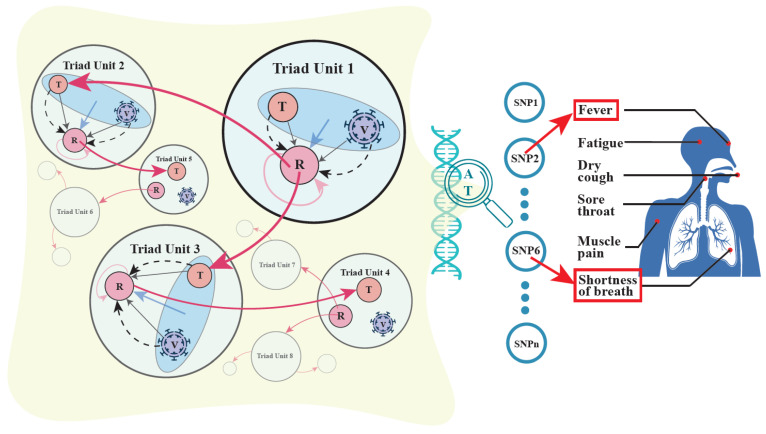
A genome-wide atlas of mobile genetic hypergraphs for COVID-19 spread in human communities. **Left panel:** Mobile hypergraphs (denoted as 1, 2, 3, …) showing the effects of pairwise and high-order genetic interactions among transmitters (T), recipients (R), and viruses (V). A hypergraph comprises a T-V-R functional triad unit (circled in light blue) that propagates COVID-19 to form an outbreak community. A red cycled arrow from R to R shows the direct genetic effect of R on its own phenotype, a black arrow from V to R shows the indirect genetic effect of the virus on the phenotype of R, and a black arrow from T to R shows the indirect genetic effect of T on the phenotype of R with the aid of the virus as a messenger. The effects of three types of horizontal pairwise epistasis, T × R, R × V, and V × T, on the phenotype of R, are shown by dot curves. Horizontal high-order epistasis is shown by a blue arrow. A unit is linked to the next through an infected person (circled in light red), who serves as a T for the former and an R for the latter. **Right Panel:** By scanning SNP 1, 2, …, (denoted by open blue circles) throughout the host genome, we can identify significant loci (e.g., SNP 2, 6, …) that affect COVID-19 spread and chart a genome-wide atlas of mobile hypergraphs. The model can discern different functions of SNPs, e.g., SNP 2 affects fever, whereas SNP 6 is responsible for the shortness of breath.

Box 1 shows the parameterization of these effects. Li et al. [74] derived a statistical algorithm for estimating and testing each of these effects. Through extensive computer simulation, they further examined the statistical properties of each estimation, which helps researchers design sampling strategies. We model the main effects as nodes, horizontal two-way epistasis effects as edges, and horizontal three-way epistasis effect as a hyperedge into a weighted hypergraph (Figure 6). The difference between such a hypergraph model and the more commonly used graph model lies in its capacity to characterize high-order interactions, i.e., interactions among three or more entities [71]. In particular, in our genetic hypergraph, we can identify how transmitter–recipient interactions are mediated by SARS-CoV-2.

If we collect the data for the individuals who contacted a transmitter but were not infected (Figure 5B), we can develop a binary model to test how genes play a role through hypergraphs in determining whether the recipients are infected. Results from this model would in turn allow us to identify specific genes that would determine the possibility of infection. A hypergraph representation can reflect both the importance of genes triggering such effects and the context dependency, in terms of how this is affected by genes from other entities. From this hypergraph, we can also characterize genetic effects, direct or indirect, horizontal two-way or horizontal three-way epistasis, as major determinants of infection.

Box 1 **Quantitative parameters that define genetic effects.**

According to Figure 5A’s sampling strategy, we would obtain genome-wide SNP data for both humans and viruses and disease outcome/phenotype data for recipients (Table S1). It is possible for mutation or methylation of viruses during their spread. Without loss of generality, we consider an SNP (with alleles A and a) at the human genome and an SNP (with two alleles A and a) at the virus genome. Each of the 18 possible tri-genome combinations among the transmitters, recipients, and viruses has a genotypic value expressed in the disease outcome (phenotype) of the recipients (Table S2). Let $\mu_{jkl}$ denote such a genotypic value due to genotype j of transmitters, genotype k of recipients, and genotype l of viruses (j,k=1 for *AA*, 0 for *Aa*, −1 for *aa*; l=1 for *B*, −1 for *b*), which can be partitioned into the following components:

$\mu_{jkl}=\mu +ja_{T}+\left(1-\left|j\right|\right)d_{T}+ka_{R}+\left(1-\left|k\right|\right)d_{R}+la_{V}\;\mathrm{Main}\;\mathrm{effects}$

$+jki_{aaTR}+j\left(1-\left|k\right|\right)i_{adTR}+\left(1-\left|j\right|\right)ki_{daTR}+\left(1-\left|j\right|\right)\left(1-\left|k\right|\right)i_{ddTR}$

$+jli_{aaTV}+\left(1-\left|j\right|\right)li_{daTV}+kli_{aaRV}+\left(1-\left|k\right|\right)li_{daRV}$ Pairwise interactions

$+jkli_{aaaTRV}+j\left(1-\left|k\right|\right)li_{adaTRV}+\left(1-\left|j\right|\right)kli_{daaTRV}+\left(1-\left|j\right|\right)\left(1-\left|k\right|\right)li_{ddaTRV}$ High-order interactions

where μ is the overall mean, $a_{T}$ and $d_{T}$ are the main additive and dominant effects of transmitters, $a_{R}$ and $d_{R}$ are the main additive and dominant effects of recipients, $a_{V}$ is the main effect of viruses; $i_{aaTR}$, $i_{adTR}$, $i_{daTR}$, and $i_{ddTR}$ are the additive x additive, additive x dominant, dominant x additive, and dominant x dominant pairwise interaction effects between transmitters and recipients, $i_{aaTV}$ and $i_{daTV}$ are the additive x virus and dominant x virus pairwise interaction effects between transmitters and viruses, $i_{aaRV}$ and $i_{daRV}$ are the additive x virus and dominant x virus pairwise interaction effects between recipients and viruses; and $i_{aaaTRV}$, $i_{adaTRV}$, $i_{daaTRV}$, and $i_{ddaTRV}$ are the additive x additive x virus, additive x dominant x virus, dominant x additive x virus, and dominant x dominant x virus high-order interactions among transmitters, recipients, and viruses.

The estimates of the magnitudes of each of these types of genetic effects may help to design more efficient drugs to control the spread of SARS-CoV-2. If direct genetic effects are significant, a drug should be designed with the capacity to directly target the genes of the recipients. If indirect genetic effects from the transmitters are significant, a drug that targets the transmitters’ genes can decrease or prevent the spread of the virus to other recipients. If indirect genetic effects from SARS-CoV-2 are significant, the viruses should be targeted. If two-way horizontal epistasis between the transmitters and recipients is significant, we need to design a drug that can decouple the transmitter–recipient genetic interaction expressed at specific loci. The existence of any three-way horizontal epistasis implies the importance of designing a drug that can destroy transmitter–recipient–virus interactions as a whole. Taken together, while conventional strategies to design vaccines aim at reducing the likelihood and degree of infection, hypergraphs will help gain new insight into the design of vaccines that control not only the infection of coronaviruses but also their spread from transmitters.

### 3.3. Mobile Hypergraphs Encapsulated in a Genome-Wide Atlas

We view a transmitter, a recipient, and the viruses that connect them as a functional triad unit. Our model proposed above can encode main genetic effects, pairwise epistatic effects, and high-order epistatic interactions among these three entities into a weighted hypergraph. The recipient of this unit may serve as a transmitter to infect another recipient, forming a new unit along with the viruses, and this process repeats until the contagion is controlled. It is expected that the behavior changes from unit to unit because the recipients and viruses vary in their genotypes. We reconstruct a hypergraph for each unit and, therefore, provide a series of dynamically changing hypergraphs, called mobile hypergraphs, in communities (Figure 6). Mobile hypergraphs can trace the topological changes in gene–gene interactions and characterize key players that determine the transmissibility of SARS-CoV-2 from person to person.

By scanning for all SNPs throughout the whole genomes of humans and viruses, we can chart a genome-wide atlas of mobile hypergraphs. From the atlas, we can identify the hotspots of genetic variants that mediates the rate and extent of coronavirus spread. As shown in Box 1, a system constituted by a transmitter, recipient, and the virus is mediated by 17 types of genetic effects each representing a different aspect of the respective genetic machinery. Thus, this atlas will be illustrated for each type of genetic effect in order to portray a comprehensive picture of the genetic mechanisms underlying COVID-19 spread.

## 4. Conclusions and Outlook

There exist great interpersonal variabilities in how humans respond to SARS-CoV-2 and how this virus transmits from person to person. Mapping the genetic components of COVID-19 contagion can not only reveal the underlying molecular mechanisms but also provide useful information for vaccine design. Existing approaches for genomic studies mainly focus on the identification of individual genes, proteins, or metabolites that are associated with the severity of COVID-19, but they do not attempt to characterize how all these entities affect the disease as a cohesive whole through reconstructing interaction networks. We propose a new computational framework for inferring maximally informative, dynamic, omnidirectional, and personalized networks (idopNetworks) from expression data [33,75]. By implementing high-dimensional statistical theory and methods, this framework can reconstruct idopNetworks at any dimension from any high or even ultrahigh dimension of data. More importantly, idopNetworks can be recovered from commonly available static data, without the need to collect more expensive but less accessible dynamic or perturbed data, making these networks a widespread tool.

We further integrate classic quantitative genetic theory and hypergraph theory to characterize how genes mediate COVID-19 transmission in human communities. This integration can systematically portray how genes from transmitters, recipients, and viruses together influence the severity and spread of COVID-19. We can further reconstruct mobile genetic hypergraphs by integrating the community structure of disease spread. By tracing the path of COVID-19 spread within and across communities, our hypergraphs can disentangle the genetic causes and consequences of each infection. Monitored results from this procedure can help the design of medications that can not only treat this disease but also block its transmission. Studies show that transmission of SARS-CoV-2 may occur during the prodromal period when those infected were mildly ill [9,10]. Thus, to impede its spread, it is of utmost importance and urgency to identify potential transmitters. Hypernetworks reconstructed from omics data may help to identify super-spreaders hidden in a population.

Several surveys have found that some individuals can more rapidly spread larger amounts of their viral load to the general population when compared to the average infected individual [76]. These individuals, known as super-spreaders, play a leading role in the epidemic network of SARS-CoV-2. Although the occurrence of super-spreaders depends on many extrinsic factors, such as frequent contacts, intrinsic factors such as coinfection with another pathogen, immune suppression, heavy viral loads, and strong virulence are thought to be crucial [76]. Previous studies have identified considerable cellular heterogeneity between super-spreaders and general transmitters [77], which suggests the possibility of distinguishing them by mapping transcriptomic, proteomic, and metabolomic variation [78,79]. Based on these omics data, our idopNetwork model can be modified to reconstruct a directed person–person network in which super-spreaders act like hub nodes. By building a system of qdODEs for characterizing how the gene expression of an individual changes over different genes, we can reconstruct gene-driven social idopNetworks that code all possible person-to-person transmissions. Thus, idopNetworks can serve as a predictor for the incoming epidemic hypernetwork from which to identify potential super-spreaders. A more stringent containment should be adopted for the super-spreaders to better control the rate and intensity of COVID-19 spread.

Our genomic and genetic physics models represent the first attempt of its kind to enhance the genetic dissection of epidemic disease. The limitations of their practical application may be overcome by equipping the statistical procedure of estimating genetic effects with the capacity to both (i) incorporate environmental components of disease outcome and (ii) allow the recipients to receive the virus from multiple transmitters. It is known that SARS-CoV-2 evolves its ability to infect humans through recombination with viruses from other host species [80], which should be incorporated for better use of the models. The incorporation of evolutionary recombination into the hypergraph model can make it biologically more meaningful and applicable. Taken together, our mobile hypergraphs, with further modifications from different perspectives, provide a conceptual lens to further our mechanistic understanding of the genetic complexities that lie behind COVID-19 and other infectious diseases.

Li et al.’s [74] model allows us to formulate a likelihood for the genotype and phenotype data collected from Figure 1A’s strategy. From this likelihood, we obtain the maximum likelihood estimates (MLEs) of $\mu_{jkl}$. By solving Equation (1) (expanded in Table S2), we estimate the MLEs of the overall mean and 17 main effects and pairwise and high-order epistatic effects. To test if these effects are collectively significant, we formulate a null hypothesis (assuming the absence of collective effects), which is compared with the alternative hypothesis (assuming the presence of collective effects) through the log-likelihood ratio (LR). The significance of collective effects can be tested by the genome-wide critical threshold determined from permutation tests. Each of these effects can be tested individually through a log-likelihood approach.

The procedure described above is used to scan all SNPs throughout the entire genomes of humans and viruses. This procedure will allow us to characterize the chromosomal distribution of significant SNPs on the human and virus genomes. Through the significance test of each effect that contributes to the genotypic value of a tri-genome combination, we can chart a genome-wide atlas of how genes govern COVID-19 infection and transmissibility. Because these effects act in different ways, representing distinct biological means, their classification is crucial for predicting and treating the disease. For example, if $d_{T}$ is significant, this implies that a transmitter carrying the heterozygous genotype *Aa* performs differently in transmissibility from those carrying homozygous genotypes *AA* and *aa*. If $d_{R}$ is significant, this implies that heterozygous recipients perform differently in disease infection from homozygous recipients. If $i_{ddTR}$ is significant, this means that the transmission of heterozygous transmitters to heterozygous recipients impacts COVID-19 infection and differs from the transmission between other genotype combinations of transmitters and recipients. The significance of high-order epistasis implies that the influence of transmitter–recipient interaction is determined by viral genes.

Our statistical mechanistic models will have immediate implications. For example, increasing studies have focused on expression changes of genes, proteins, and metabolites in COVID-19-infected individuals [10]. By applying our models to these studies, we will not only characterize key individual entities but also unveil how each entity interacts with every other entity to determine COVID-19 symptoms. From these interactions, we can enhance the efficiency of drug design by targeting interactive genes, proteins, and metabolites. Additionally, when COVID-19 studies are expanded to the population level, our genetic model can detect and map important DNA variants and their interactions that take place among recipients, transmitters, and viruses. All this information can help build a predictive model for COVID-19 risk and spread.

## Data Availability

Computer code can be available upon request from the corresponding author.

## Supplementary Materials

The following supporting information can be downloaded at: https://www.mdpi.com/article/10.3390/cells11010080/s1, Table S1: Data structure of SNPs and disease outcomes/phenotypes for transmitters, recipients, and the virus from the sampling strategy as shown in Figure 1A. Table S2: Quantitative genetic effects of genes from transmitters (T), recipients (R), and the virus (V), which can be estimated from Table S1. Figure S1: Fine-grained networks (for module M3 composed of 463 proteins), with a comparison between control and COVID-19 patients, visualized by Voronoi treemaps where each polygon area (node) is represented by a protein (with its name shown). The color metric is proportional to the overall expression level of this protein. Activation and inhibition are denoted by arrowed red-color and blue-color lines, respectively, with the thickness of lines being proportional to the strength of protein interactions.
