# Supplementary material for: The Genomic Physics of COVID-19 Pathogenesis and Spread
Source: Cells. 2021 Dec 28;11(1):80. doi: 10.3390/cells11010080 (PMC8750765; doi:10.3390/cells11010080)
Supplement: Supplementary file 1 [file cells-11-00080-s001.zip › cells-1488559-supplementary.pdf]

Control

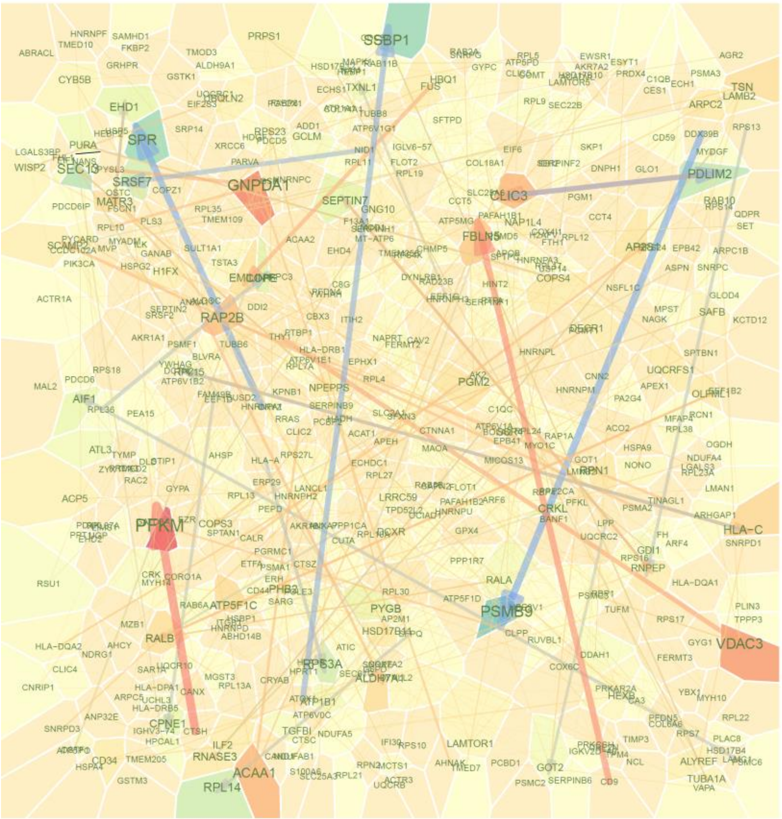

COVID−19

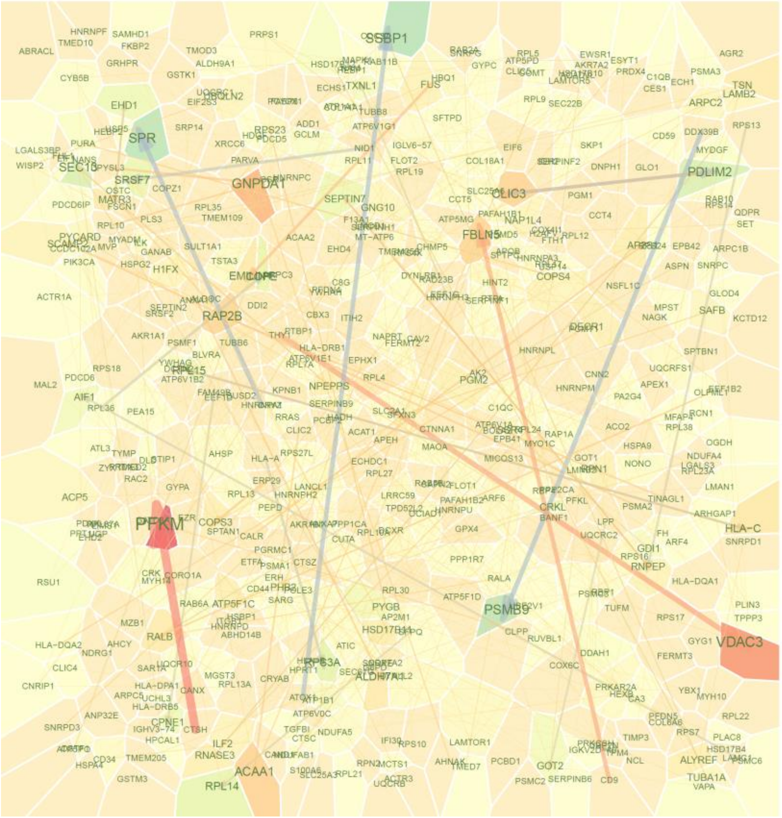

Up−regulation

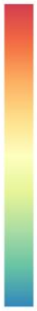

Down−regulation

Figure S1 Fine-grained networks (for module M3 composed of 463 proteins), with a comparison between control and COVID-19 patients, visualized by Voronoi treemaps where each polygon area (node) is represented by a protein (with its name shown), with the color metric being proportional to the overall expression level of this protein. Activation and inhibition are denoted by arrowed warm-color and cold-color lines, respectively, with the thickness of lines being proportional to the strength of protein interactions.

Table S1 Data structure of SNPs and disease outcomes/phenotypes for transmitters, recipients, and the virus from the sampling strategy as shown in Fig. 1A.

		Host SNP					Virus Gene			Phenotype		
		1		…	*m*							
Unit	Host T→R	T	R	…	T	R	1	…	*q*	1	…	*p*
1	0→1	0	AA	…	0	Aa	A	…	a	y_{11}	…	y_{p1}
2	1→2	AA	Aa	…	Aa	AA	a	…	a	y_{12}	…	y_{p2}
3	1→3	AA	aa	…	Aa	aa	A	…	A	y_{13}	…	y_{p3}
4	1→4	AA	AA	…	Aa	Aa	a	…	a	y_{14}	…	y_{p4}
5	1→5	AA	Aa	…	Aa	aa	a	…	A	y_{15}	…	y_{p5}
6	2→12	Aa	aa	…	AA	AA	a	…	A	y_{16}	…	y_{p6}
7	2→13	Aa	Aa	…	AA	Aa	A	…	a	y_{17}	…	y_{p7}
8	2→14	Aa	AA	…	AA	aa	a	…	a	y_{18}	…	y_{p8}
9	2→15	Aa	Aa	…	AA	aa	A	…	A	y_{19}	…	y_{p9}
10	3→10	aa	aa	…	aa	AA	a	…	A	y_{110}	…	y_{p10}
11	3→11	aa	AA	…	aa	Aa	A	…	a	y_{111}	…	y_{p11}
12	4→8	AA	aa	…	Aa	aa	a	…	A	y_{112}	…	y_{p12}
13	4→9	AA	aa	…	Aa	Aa	A	…	A	y_{113}	…	y_{p13}
14	5→6	Aa	Aa	…	aa	aa	a	…	A	y_{114}	…	y_{p14}
15	5→7	Aa	AA	…	aa	Aa	A	…	a	y_{115}	…	y_{p15}
16	6→17	Aa	aa	…	aa	aa	a	…	A	y_{116}	…	y_{p16}
17	7→18	AA	aa	…	Aa	aa	A	…	A	y_{117}	…	y_{p17}
18	8→19	aa	aa	…	aa	AA	a	…	A	y_{118}	…	y_{p18}
19	9→20	aa	Aa	…	Aa	aa	A	…	a	y_{119}	…	y_{p19}
20	14→16	AA	aa	…	Aa	AA	A	…	a	y_{120}	…	y_{p10}

The data include three parts: (1) Host genotypes for transmitters (T) and recipients (R) at *m* SNPs, (2) viral genotypes at *q* loci, and (3) disease outcome phenotypic values for *p* traits for 20 recipients. Host genotypes are displayed in terms of T→R pairs, where → denotes the direction of transmission from T to R in a functional unit.

Table S2 Quantitative genetic effects of genes from transmitters (T), recipients (R), and the virus (V), which can be estimated from Table S1.

No.	Cross-genome Combination: Genotype T	R	V	Genotypic Value
1	AA	AA	A	$\mu_{111} = \mu + a_T + a_R + a_V + i_{aaTR} + i_{aaTV} + i_{aaRV} + i_{aaaTRV}$
2	AA	AA	a	$\mu_{110} = \mu + a_T + a_R - a_V + i_{aaTR} - i_{aaTV} - i_{aaRV} - i_{aaaTRV}$
3	AA	Aa	A	$\mu_{121} = \mu + a_T + d_R + a_V + i_{adTR} + i_{aaTV} + i_{daRV} + i_{adaTRV}$
4	AA	Aa	a	$\mu_{120} = \mu + a_T + d_R - a_V + i_{adTR} - i_{aaTV} - i_{daRV} - i_{adaTRV}$
5	AA	aa	A	$\mu_{131} = \mu + a_T - a_R + a_V - i_{aaTR} + i_{aaTV} - i_{aaRV} - i_{aaaTRV}$
6	AA	aa	a	$\mu_{132} = \mu + a_T - a_R - a_V - i_{aaTR} - i_{aaTV} + i_{aaRV} + i_{aaaTRV}$
7	Aa	AA	A	$\mu_{211} = \mu + d_T + a_R + a_V + i_{daTR} + i_{daTV} + i_{aaRV} + i_{daaTRV}$
8	Aa	AA	a	$\mu_{212} = \mu + d_T + a_R - a_V + i_{daTR} - i_{daTV} - i_{aaRV} - i_{daaTRV}$
9	Aa	Aa	A	$\mu_{221} = \mu + d_T + d_R + a_V + i_{ddTR} + i_{daTV} + i_{daRV} + i_{ddaTRV}$
10	Aa	Aa	a	$\mu_{222} = \mu + d_T + d_R - a_V + i_{ddTR} - i_{daTV} - i_{daRV} - i_{ddaTRV}$
11	Aa	aa	A	$\mu_{231} = \mu + d_T - a_R + a_V - i_{daTR} + i_{daTV} - i_{aaRV} + i_{daaTRV}$
12	Aa	aa	a	$\mu_{232} = \mu + d_T - a_R - a_V - i_{daTR} - i_{daTV} - i_{aaRV} + i_{daaTRV}$
13	aa	AA	A	$\mu_{311} = \mu - a_T + a_R + a_V - i_{aaTR} - i_{aaTV} + i_{aaRV} - i_{aaaTRV}$
14	aa	AA	a	$\mu_{312} = \mu - a_T + a_R - a_V - i_{aaTR} + i_{aaTV} - i_{aaRV} + i_{aaaTRV}$
15	aa	Aa	A	$\mu_{321} = \mu - a_T + d_R + a_V - i_{adTR} - i_{aaTV} + i_{daRV} - i_{adaTRV}$
16	aa	Aa	a	$\mu_{322} = \mu - a_T + d_R - a_V - i_{adTR} + i_{aaTV} - i_{daRV} + i_{adaTRV}$
17	aa	aa	A	$\mu_{331} = \mu - a_T - a_R + a_V + i_{aaTR} - i_{aaTV} - i_{aaRV} + i_{aaaTRV}$
18	aa	aa	a	$\mu_{332} = \mu - a_T - a_R - a_V + i_{aaTR} + i_{aaTV} + i_{aaRV} - i_{aaaTRV}$

Genetic effect parameters

$\mu_{...}$'s are genotypic values of cross-genome genotypes among transmitters, recipients, and the virus, which are partitioned into the population mean (μ), main additive genetic effects (a_T, a_R, a_V), main dominant genetic effects (d_T, d_R), pairwise horizontal epistatic effects (i_{aaTR}, i_{adTR}, i_{daTR}, i_{ddTR}, i_{aaTV}, i_{daTV}, i_{aaRV}, i_{daRV}), and three-way horizontal epistatic effects (i_{aaaTRV}, i_{adaTRV}, i_{daaTRV}, i_{ddaTRV}).

Advantages of the hypergraph model

Main additive and dominant genetic effects, a_R and d_R, describe how the gene from recipients directly affects their own phenotypes, whereas main additive and dominant genetic effects, a_T and d_T, describe how the gene from transmitters indirectly affects the phenotypes of recipients and main genetic effect, a_V, describes how the gene from the virus indirectly affects the phenotypes of recipients. Pairwise and high-order epistatic effects describe how genes from transmitters, recipients, and the virus interact with each other to affect the phenotypes of recipients. A genetic hypergraph is reconstructed by coding main **direct and indirect** additive and dominant genetic

effects as nodes, pairwise epistatic effects as edges, and three-way epistatic effects as hyperedges into a hypergraph.

Traditional quantitative genetic models can only estimate effect parameters a_R and d_R; i.e., direct genetic effects of genes carried by infected individuals on their own phenotypes, failing to characterize indirect genetic effects of genes (a_T and d_T and a_V) carried by transmitters and the virus on the phenotype of recipients. None of these models can estimate the effects of horizontal epistasis on the phenotype of recipients. If quantitative genetic models are integrated with classic graph theory (although there is no such an effort made in the literature), then we can only characterize pairwise horizontal epistasis, with high-order horizontal epistasis to be unrevealed.
